# A wireless and handheld optical palpation imaging probe for use in breast-conserving surgery

**DOI:** 10.1063/5.0323681

**Published:** 2026-05-05

**Authors:** Rhys Jones, Renate Zilkens, Ankit Bharakhda, Mireille Hardie, Christobel M. Saunders, Qi Fang, Brendan F. Kennedy

**Affiliations:** 1 BRITElab, Harry Perkins Institute of Medical Research, QEII Medical Centre, Nedlands, Western Australia 6009, Australia and Centre for Medical Research, The University of Western Australia, Perth, Western Australia 6009, Australia; 2 Department of Electrical, Electronic & Computer Engineering, School of Engineering, The University of Western Australia, Perth, Western Australia 6009, Australia; 3 Division of Surgery, Medical School, The University of Western Australia, Crawley, Western Australia 6009, Australia; 4 Division of Pathology and Laboratory Medicine, Medical School, The University of Western Australia, Perth, Western Australia 6009, Australia; 5 Department of Surgery, Melbourne Medical School, The University of Melbourne, Parkville, Victoria 3010, Australia; 6 The Royal Melbourne Hospital, 300 Grattan Street, Parkville, Victoria 3050, Australia; 7 Institute of Physics, Faculty of Physics, Astronomy and Informatics, Nicolaus Copernicus University in Toruń, Grudziadzka 5, 87-100 Torun, Poland; 8 Institute of Advanced Studies, Nicolaus Copernicus University in Torun, Grudziadzka 5, 87-100 Torun, Poland

## Abstract

We present a wireless and handheld optical elastography probe aimed toward improving intraoperative discrimination between malignant and benign tissue in breast-conserving surgery. If successful, this probe can contribute to reducing close or positive margins and, therefore, subsequent re-excisions. The probe visualizes mechanical contrast between tumor and surrounding benign tissue in excised human breast specimens using stereoscopic optical palpation, in which the deformation of a compliant silicone layer is measured by two parallel cameras to infer surface stress, where variations in stress correspond to differences in the mechanical properties of the underlying tissue. Wireless operation is achieved using a Wi-Fi transceiver to transmit images at 15 fps to a laptop for processing. To enhance image quality, we incorporate computational optical palpation, which utilizes finite element analysis to provide a more accurate mechanical model of the deformation of the compliant layer. This approach yields a twofold improvement in spatial resolution, from 1034 to 512 *μ*m, and a 55% increase in stress contrast in a structured silicone phantom. In a preliminary study on four excised human breast tissue samples, we demonstrate that the probe can identify tumor and distinguish between benign tissue types, including adipose tissue, stroma, and potentially ducts.

## I. INTRODUCTION

Breast-conserving surgery (BCS) is the primary surgical treatment for early-stage breast cancer, a disease that impacts over two million women globally each year.^1–3^ The primary objective of BCS is to excise tumor with a surrounding margin of healthy tissue. This procedure is typically followed by adjuvant therapy, such as radiotherapy or chemotherapy. However, incomplete tumor excision remains a significant clinical challenge, with 15%–20% of patients requiring reoperation due to positive or close margins.^4,5^ These repeat surgeries not only delay follow-up treatment but also impose substantial physical, psychological, and economic burdens on patients.^6–8^

Accurate intraoperative detection of tumor is therefore critical to achieving complete tumor removal while minimizing unnecessary tissue excision. Clinical palpation, the surgeon's sense of touch, remains an essential component of the standard of care. In this technique, the surgeon uses intrinsic differences in mechanical properties between stiff tumor and surrounding softer benign tissue. However, while useful, clinical palpation has inherently low sensitivity, is subjective and, ultimately, does not adequately reduce the re-excision rate.^9^ In an effort to address this, several intraoperative margin assessment techniques have been proposed and implemented clinically, including pathology-based methods such as frozen section analysis and imprint cytology,^10–12^ and imaging modalities like specimen radiography.^13^ However, none of these techniques have adequately reduced the re-excision rate. In addition, these techniques have a range of challenges, including long turnaround times, cost, and dependency on additional personnel.^14,15^ A range of biophotonics techniques have also been proposed, including optical spectroscopy^16^ and optical coherence tomography (OCT);^17,18^ however, these techniques often involve complex and expensive imaging systems that require advanced image processing algorithms and have yet to demonstrate the clinical value required for widespread uptake. As a result, many surgeons still often rely on clinical palpation for intraoperative assessment.

Leveraging the intrinsic difference in mechanical properties between tumor and benign tissue, as done in clinical palpation, optical elastography techniques have been developed for intraoperative cancer detection.^19,20^ In particular, optical coherence elastography (OCE) employs OCT as the imaging modality to map tissue deformation in response to an induced mechanical load and utilizes continuum mechanics to generate images of a mechanical property, such as Young's modulus.^21,22^ In variants of OCE based on compressive loading, studies on excised tissue have demonstrated high diagnostic accuracy (>90%).^23,24^ In addition, handheld OCE probes have been developed to provide direct, *in vivo* assessment of residual tumor in the surgical cavity, with the goal of providing a more practical and accurate solution that integrates more effectively with the surgical workflow.^25–28^ However, these approaches are expensive and complex and have relatively low acquisition speeds (several seconds), making them susceptible to motion artifacts. Additionally, OCE probes require electrical cables and optical patch cords that cross the sterile surgical field, further complicating their routine use in operating theaters.^29^ In optical palpation, another variant of optical elastography based on compressive loading, a compliant silicone layer is placed between the tissue and the imaging window, which acts as a compression plate. Under compression, the layer deformation is dependent on the mechanical properties of the underlying tissue, with greater deformation above stiffer regions. Two-dimensional (2D) stress maps are generated by first mapping the strain in the layer in each lateral position and then estimating stress from the pre-characterized stress–strain response of the layer. In initial demonstrations of optical palpation, the layer deformation was estimated using OCT.^30^ However, more straightforward and cost-effective approaches based on interferometry^31^ and digital cameras^32,33^ have since been developed. In particular, stereoscopic optical palpation (SOP) is an approach that utilizes two parallel digital cameras to measure layer deformation.^33^ Importantly, SOP uses a layer with an embedded plane of phosphorescent microparticles that emit green light under ultraviolet (UV) illumination and are mostly transparent under visible light. Incorporating both visible light and UV light emitting diodes (LEDs) in the setup allows SOP to sequentially capture white light photographs and images of the UV illuminated particle plane, which are used to calculate mechanical stress at the tissue surface,^33^ as described in Sec. V A. The white light photographs facilitate accurate co-registration with histology and provide important visual context for surgeons. In a diagnostic accuracy study, a benchtop SOP system demonstrated a sensitivity of 86% and a specificity of 83%, approaching the accuracy needed for intraoperative detection.^34^ Importantly, the small footprint, video rate acquisition, and straightforward implementation make SOP especially suitable for development in a handheld format for *in vivo* use compared to other forms of optical elastography.

In this study, we describe a wireless and compact handheld SOP probe, which is designed for rapid intraoperative breast cancer detection. The device is optimized for portability and ease of use, featuring a rechargeable battery and wireless data transmission via a Wi-Fi transceiver. We validate the probe's performance on silicone phantoms containing embedded stiff inclusions, demonstrating its ability to visualize mechanical contrast. Additionally, we adapt computational optical palpation, a technique that increases stress contrast and spatial resolution by utilizing finite element analysis (FEA), to account for boundary conditions and to extract the stress at the layer surface.^35^ Finally, we demonstrate the imaging performance of the wireless probe on freshly excised breast tissue specimens, confirming the ability to differentiate malignant from benign tissue through validation by histology.

## II. RESULTS

### A. Wireless handheld probe design outcomes

The first step was to establish how the probe would be used during surgery and, therefore, the clinical requirements for the probe. After consultation with breast surgeons, conducted as free-form interviews touching on the topics of handheld probe use, breast-conserving surgery, equipment acquisition, display media, and ergonomics, we determined the following key design requirements:

• an ergonomic handheld format;
• a field of view (FOV) of at least 6 × 6 mm;
• wireless operation;
• a minimum run time of 1 h; and
• material costs less than 1500 United States Dollars (USD).

**FIG. 1.**
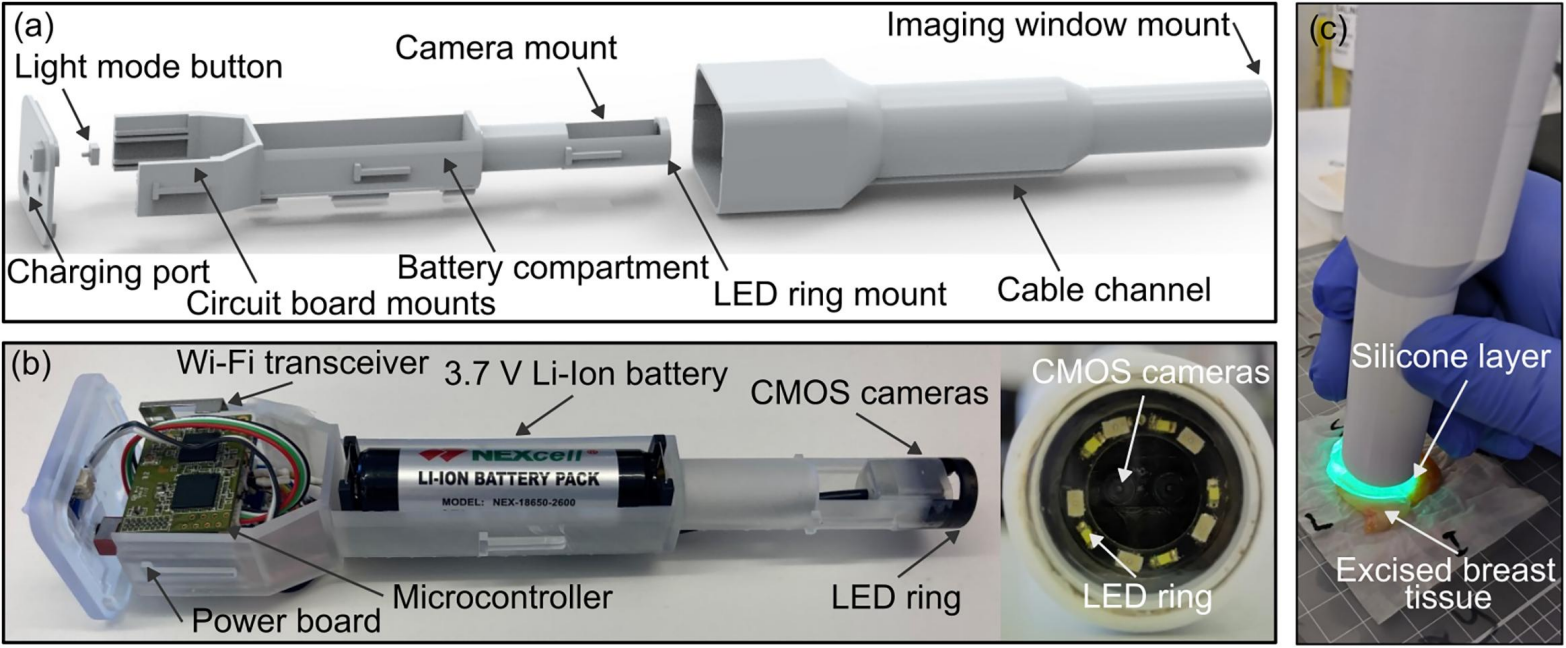
Wireless handheld probe schematic and components. (a) Digital model of the 3D printed components of the probe designed and visualized using Solid Edge. (b) Assembled and labeled view of the interior probe components. (c) Photograph of the handheld probe and compliant layer in use on an excised human breast tissue sample under UV illumination.

The probe designed in this project, pictured in Fig. 1, and described in additional detail in the supplementary material, met each of these requirements as described below.

*Ergonomic handheld format*: The probe comprises two sections, an interior framework to which components are mounted and an exterior case. The interior framework was 3D printed using a stereolithography apparatus (SLA) printer (Formlabs Form2 with clear v4 resin) and the power and microcontroller boards were mounted to the casing as shown in Fig. 1(a). The Wi-Fi module was then mounted via a 16-pin attachment to the microcontroller board. The interior framework also provides a ring on which to mount the LEDs, a section for a custom camera mount to be fastened to, a battery compartment, and “T” shaped connectors to attach to the outer case, as illustrated in Fig. 1(b). The outer case was 3D printed using a Prusa i3 Mk3 with Prusa polylactic acid (PLA) filament with a thinner probe tip compared to the middle and back sections to provide precision grip, and the cable channel provides tactile feedback to determine probe orientation when in use, as shown in Fig. 1(c). The assembled probe fits inside a rectangular prism with dimensions 210 × 49 × 43 mm and weighs ∼170 g.

*Field of view*: The probe was designed to acquire images under both UV and white light illuminations, providing a field-of-view of ∼8 × 10 mm. A 9 mm inner diameter ring containing five 365 nm UV LEDS (Kingbright ATS2012UV365) and five white light LEDs (Inolux IN-S63AT5UW) was used to illuminate the sample and a button at the back of the probe was used to switch between modes. Images were acquired using two complementary metal–oxide–semiconductor (CMOS) cameras (MISUMI, 1/9″ Color CMOS 720p Advance Dual Camera), with centers spaced 5.5 mm apart and the camera plane was placed ∼16 mm from the front of the imaging window. The end of the interior framework was coated with black ink to mitigate fluorescence emissions from the clear v4 resin material under UV illumination, which would otherwise add unwanted background signal to the camera images. Filament-based 3D printing with PLA was used to avoid fluorescence in the outer case, as the SLA printer's finer resolution was not required.

*Wireless operation*: The dual camera system collects frames from each sensor and merges them side by side to create a single data stream, which is sent to a computer via a USB Video Class (UVC) Wi-Fi Wireless AP Router (MISUMI) at 15 frames per second (fps).

*Run time*: The device is powered by a 3.7 V, 2600 mAh lithium-ion battery, lasting 1.5–2 h depending on LED use, with a peak power consumption of 5.9 W. The power board regulates the battery voltage via a buck-boost converter, handles the device's on–off switch, and provides a charging circuit for the battery. The microcontroller board handles the logic for the button switching LED modes and includes a boost converter for the LEDs. A micro-USB connector and charging circuit were incorporated in the probe to allow the device to be recharged, with a full recharge time of 4 h.

*Material costs*: The total cost of materials used in the probe was USD 1188. This included the camera system (USD 890), Wi-Fi transceiver (USD 71), battery and housing (USD 17), resin and filament materials (USD 3), custom circuit board printing (USD 100), and other electronic components (USD 107).

### B. Silicone phantom

**FIG. 2.**
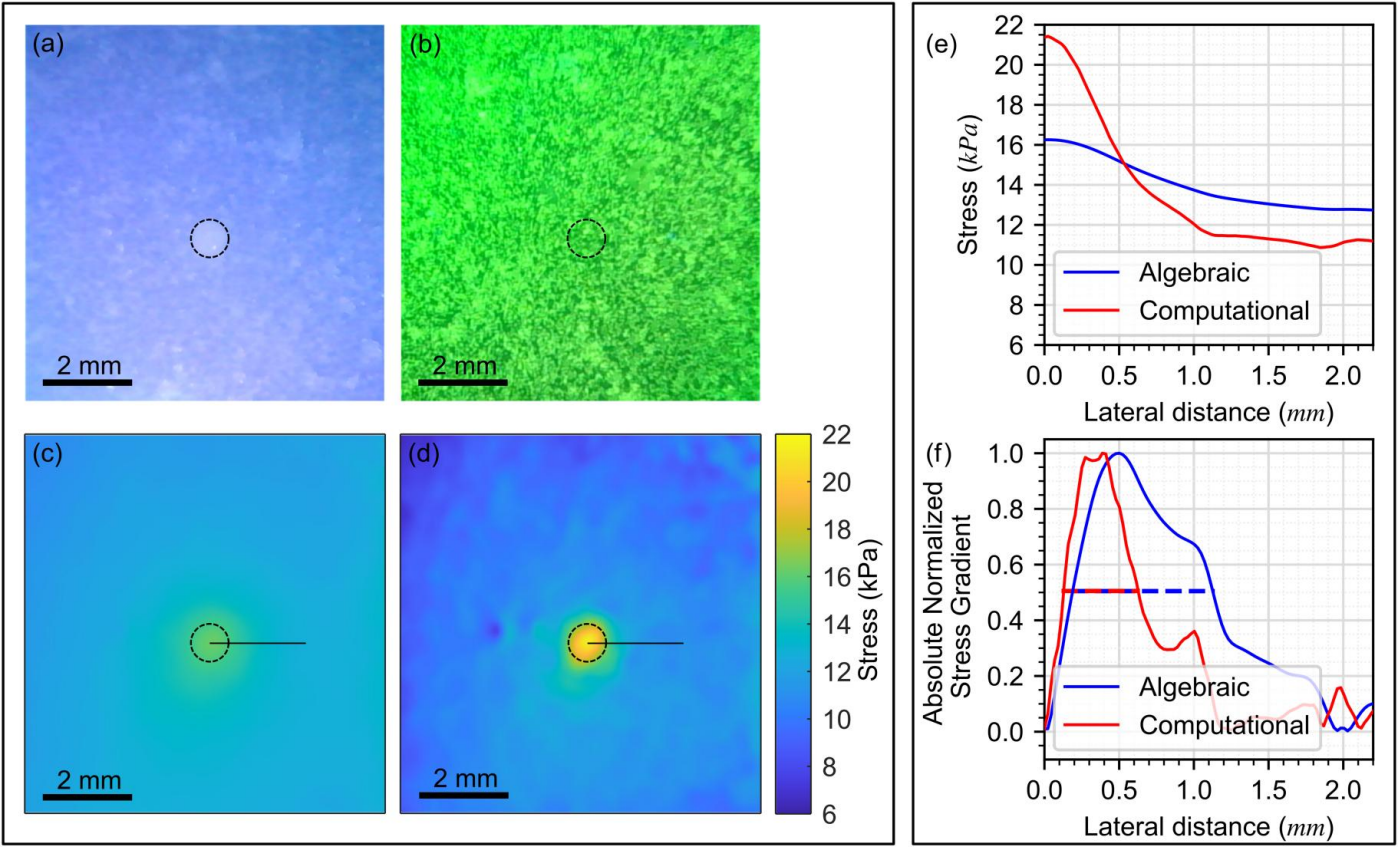
Wireless handheld SOP probe results on a structured phantom, with the 0.8 mm diameter inclusion boundary marked by a black dashed circle in (a)–(d). (a) Probe left camera image under white light illumination. (b) Probe left camera image of the phosphorescent particles under UV illumination. (c) Stress map of the inclusion phantom derived from the algebraic mechanical model. (d) Stress map of the inclusion phantom derived using computational optical palpation. (e) Stress plots extracted from (c) and (d) along the horizontal black lines, showing the change in stress from the center of the inclusion to the background. (f) Absolute value of the normalized gradients of the stress plots in (e), with dashed lines marking the full width at half maximum of each peak, corresponding to the resolution from each method.

**FIG. 3.**
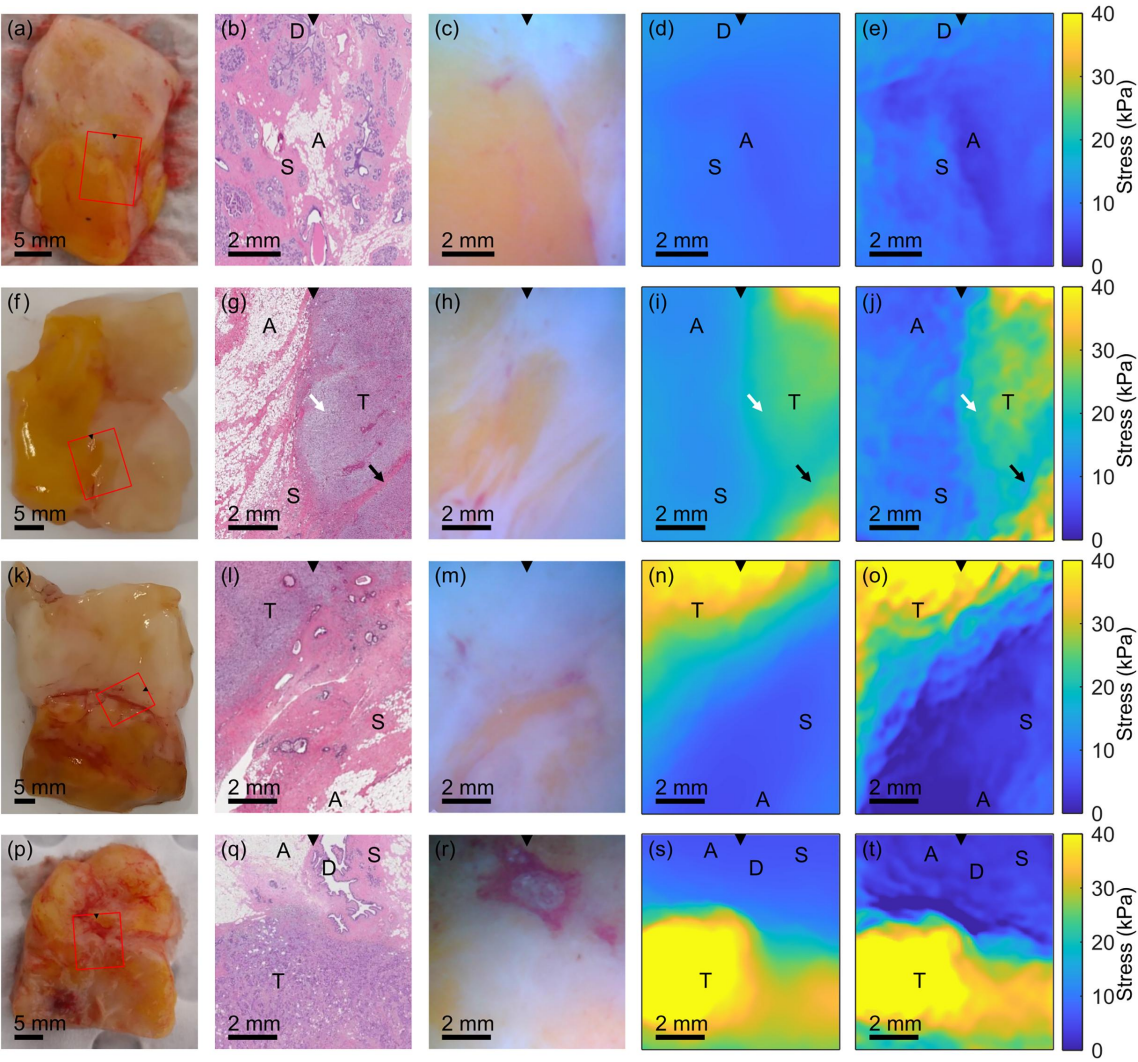
Wireless and handheld SOP probe results from four excised human breast tissue samples. (a), (f), (k), (p) Macroscopic photographs of the excised tissue, taken with a DSLR camera, where the red rectangle indicates the region imaged by the probe, with the black triangle denoting the top of the image. (b), (g), (l), (q) Histology of the region imaged using the probe. (c), (h), (m), (r) Probe left camera image under white light illumination. (d), (i), (n), (s) Stress map derived from the algebraic mechanical model. (e), (j), (o), (t) Stress map derived from computational optical palpation. Labels have been used to denote the locations of different tissue types, where A = adipose tissue, S = stroma, D = duct, T = tumor.

We evaluated the handheld probe on a silicone phantom with a stiff inclusion that mimics tissue mechanical properties,^36^ described in Sec. V C, enabling quantitative assessment of imaging metrics. In Fig. 2(a), the visible light image is presented, with the inclusion highlighted by a black dashed circle. While the scattering from the phosphorescent particles is very low under white light illumination, they are visible in Fig. 2(a) because of the low scattering of the sample combined with the automatic exposure and contrast adjustments of the dual camera system. Importantly, as can be seen in the tissue results in Fig. 3, these particles are not visible when there is a highly scattering tissue sample beneath the layer. Figure 2(b) shows the corresponding image of the phosphorescent particle pattern under UV illumination. There is no visible contrast between the inclusion and the background; however, the location of the inclusion is known from the corresponding white light image and is highlighted with a black dashed circle. In Figs. 2(c) and 2(d), the algebraic and computational optical palpation images are presented, respectively, with the inclusion visible in both cases. The algebraic approach refers to using the probe's measurements of layer deformation to estimate bulk axial strain, then converting strain to stress using the pre-characterized mechanical model of the layer,^33^ as presented in Sec. V A. In addition, details of the computational optical palpation method are provided in Sec. V B. In Fig. 2(e), we present the variation in stress in the lateral direction from the center of the inclusion to the background material, as indicated by the black line in Figs. 2(c) and 2(d). A noticeable increase in contrast is visible when the computational method is employed. Defining stress contrast as the stress at the center of the inclusion divided by the stress in the background,^37^ from the plot in Fig. 2(e), we observe a stress contrast of 1.28 using the algebraic method, compared to 1.97 using the computational method, a ∼55% increase. In Fig. 2(f), to determine stress resolution, we present the magnitude of the normalized gradient of the stress curves in Fig. 2(e). The resolution in each case is calculated as the full width at half maximum of the stress gradient, as we have shown previously.^37^ The stress resolution improved from 1034 *μ*m using the algebraic approach to 512 *μ*m using the computational method.

### C. Excised human breast tissue

In Fig. 3, we present results from four freshly excised human breast tissue specimens, ranging from benign tissue to dense tumor. Figure 3(a) shows a macroscopic photograph of a benign specimen acquired with a digital single-lens reflex (DSLR) camera, where a red rectangle highlights the area imaged by the SOP probe. In Fig. 3(b), a region of histology is shown that matches the area imaged by the SOP probe. The co-registration was facilitated by the white light photograph acquired using the probe, presented in Fig. 3(c). From the histology image depicted in Fig. 3(b), the tissue imaged by the probe contains a central region of adipose tissue, surrounding stroma and ducts, labeled “A,” “S,” and “D,” respectively in Figs. 3(b), 3(d), and 3(e). In the algebraic optical palpation image in Fig. 3(d), a slight decrease in stress is visible in the region corresponding to adipose tissue, indicating that it is softer than surrounding tissue, as expected.^38^ There are also slight variations in stress in the surrounding tissue, which may correspond to variations in mechanical properties between ducts and stroma.^39^ The computational optical palpation image presented in Fig. 3(e) demonstrates substantial improvement in image contrast, with a greater contrast between the adipose tissue and surrounding tissue, and a sharper transition visible between regions, as expected from the increased spatial resolution. There is also increased texture in the stroma and duct regions, potentially resulting from an increase in stress contrast between these tissue types after applying the computational method.

Figures 3(f)–3(j) present results from a spindle cell carcinoma case, with Fig. 3(f) presenting the macroscopic photograph with the probe imaging FOV indicated by a red rectangle. Comparing Figs. 3(g) and 3(i), we see low stress regions from the algebraic optical palpation image matching adipose tissue and stromal tissue in histology, and high stress regions matching tumor, labeled “T.” The computational optical palpation image in Fig. 3(j) provides increased resolution and contrast and presents a sharper transition between tissue regions. It also reveals mechanical contrast within the tumor region. The relatively low stress on the center left of the tumor region likely corresponds to an area containing myxoid stroma, indicated by the lighter shade in the histology in Fig. 3(g) and labeled with a white arrow, potentially due to replacement of an organized collagen network by the myxoid matrix.^40^ At the bottom right of the histology image, a band of non-cancerous stroma is visible running diagonally through the tumor, which may correspond to the low stress band in the same location in the computational image in Fig. 3(j). A black arrow marks the location of this feature in the histology image, the algebraic stress image, and the computational stress image.

Figures 3(k)–3(o) present results from a second specimen from the spindle cell carcinoma case, taken from a different boundary region of the large tumor. Comparing the algebraic and computational optical palpation images in Figs. 3(n) and 3(o) with the histology image in Fig. 3(l), we again observe a correspondence between low stress regions and adipose tissue and stroma, and high stress regions and tumor. Surface topology variations may have influenced a reduction in apparent coincidence between tumor regions and high stress, as evidenced by a need for a deeper plane recut during histological processing of this sample, as the first plane was missing tissue in large parts of the region.

Finally, Figs. 3(p)–3(t) present results from a specimen containing invasive ductal carcinoma (IDC). The algebraic and computational stress images in Figs. 3(s) and 3(t) show a clear boundary between tumor and surrounding adipose, stromal and ductal tissue, matching the histology image in Fig. 3(q). The left area within the tumor exhibits very high stress that does not correspond to any identifiable features in the histology. This may reflect a contribution to the measured stress from local surface topology variations or by subsurface tissue structures not captured in the histological plane, for example, the stiff tumor extending deeper into the sample in that region. However, similar to previous results, the stress images maintain a sharp transition at the tumor boundary.

## III. DISCUSSION

In this paper, we present images of phantoms and excised human breast tissue acquired using a novel, wireless, handheld optical elastography probe. In a proof-of-principle study on excised human breast tissue, the probe demonstrated the capability to distinguish between tumor and surrounding, uninvolved tissues. Importantly, the image quality presented using the algebraic mechanical model is comparable to a previous diagnostic accuracy study conducted using a benchtop SOP system on tissue samples from 48 patients, which demonstrated a sensitivity and specificity of 86% and 83%, respectively.^34^ Furthermore, by incorporating computational optical palpation in the signal processing, we demonstrated enhanced stress contrast and resolution over the previous benchtop study. These enhancements may allow for the detection of smaller regions of tumor which indicates the potential for the probe to provide improved diagnostic performance in future studies.

The cost of the components used to manufacture our probe was USD 1188. Importantly, as the components are standard consumer electronics, the cost could be significantly reduced in mass production, lowering the barrier to widespread uptake if the clinical efficacy of the device is established.

Despite its advantages, a number of improvements could be made to the electrical design of the probe to facilitate larger clinical studies. For example, wireless charging pads could be incorporated to facilitate ease of sterilization and more practical use in surgical theaters. In addition, although our probe exceeded the minimum required run time of 1 h, to avoid the need to turn the device on and off repetitively between use, it may be necessary to extend the battery life. This could be achieved by using a larger capacity battery and more efficient components and by more robust manufacturing of the electrical connections in the probe to reduce losses.

In our results, we observe increased texture in the computational stress images, relative to the algebraic images. Our results suggest that this texture is primarily driven by the ability of the computational approach to resolve micro-scale mechanical heterogeneity that is not visualized in the algebraic method. This is supported by the close spatial correspondence between features in the computational images and the tissue features observed in co-registered histology. This is especially apparent in Fig. 3(e), where the lower stress region in the computational image corresponds to a region of soft adipose tissue in histology (marked by the letter A), surrounded by stiffer stroma and ductal tissue, a detail that was not visible in the algebraic image. Similarly, in Fig. 3(j), the computational image shows a sharper edge between tumor and benign tissues, and a band of stroma seen in histology (marked by a black arrow) can be located within the computational image as a lower stress region within the tumor. However, this heightened sensitivity is accompanied by a vulnerability to measurement noise at the sample-layer interface. Because the computational method relies on position measurements at the surface of the layer–sample interface, small irregularities stemming from surface roughness, non-uniformities in the phosphorescent particle distribution, or localized friction can be amplified into large variations in stress at the surface that do not correspond to the underlying mechanical properties of the sample. This is evidenced by the residual texture observed in the homogeneous silicone phantom image in Fig. 2(d). Although this sensitivity to noise necessitates cautious interpretation of images, the visualization of previously obscured tissue features supports the use of the computational approach.

While handheld operation of the probe facilitates straightforward clinical use, with our current probe design, the force applied to the tissue is heavily dependent on the angle of the user's hand relative to the sample. In this initial demonstration on excised tissue, we minimized the angle between the tissue surface and the probe. However, a future design of the probe would need to consider more robust use by clinicians *in vivo* during surgery, where it is more challenging to control this angle. One solution is to design the probe tip to be spherical, making it more resistant to angled contact.

Similar to the benchtop implementation of SOP,^33^ the layer was physically separate from the probe such that it was placed on the tissue first, followed by the user manually applying load to the tissue. For more practical use in surgery, the layer could be affixed to the probe tip using several methods outlined in previous OCE studies.^28,41^ In the case where the layer is bonded to the surface of the imaging window, computational optical palpation can be adapted to account for the changes in boundary conditions without loss of accuracy.

Analogous to clinical palpation, stress maps are a qualitative measurement of mechanical properties and do not explicitly measure the depth of mechanical features from the surface. It may be possible to quantify mechanical properties using our probe by measuring the lateral strain in the sample, using the white light imaging functionality to track the deformation of visible features and combine this with stress measurements from the layer to estimate elasticity. To extract depth information, in the related field of tactile imaging, inverse methods have been proposed to reconstruct the 3D mechanical properties from stress measurements either at a range of loads^42^ or using neural networks.^43,44^ Future work could include adapting these methods to make them computationally efficient for real-time use during surgery.

Beyond breast cancer, there are a number of scenarios where clinical palpation is used, such as in biopsy guidance or the intraoperative assessment of margins in prostate cancer surgery,^45,46^ suggesting that SOP may have broader clinical applications. More generally, the probe could be applied to many cases where cancer is surrounded by softer tissue, such as in head and neck cancer^47,48^ or brain cancer.^49^ Optical palpation has also demonstrated potential in burn scar assessment in remote settings.^50^

## IV. CONCLUSION

In this paper, we have presented a wireless handheld optical elastography probe, using SOP, a cost-effective and small footprint imaging modality, capable of mapping stress at the tissue surface. Using computational optical palpation, an FEA-based approach, to more accurately estimate stress from the measured deformation of the layer, we demonstrated an improvement in spatial resolution from 1034 to 512 *μ*m and an increase in contrast of ∼55%. By imaging excised human breast specimens, we have demonstrated that the probe can visualize the mechanical contrast between invasive tumor and benign tissue, with the computational method allowing distinction between different types of soft benign tissue. We believe that key features of our wireless and handheld probe make it well-suited for clinical translation, including dual-mode stress and white light imaging, low material cost, and improved imaging performance when using the computational method.

## V. METHODS

### A. Stress map generation

**FIG. 4.**
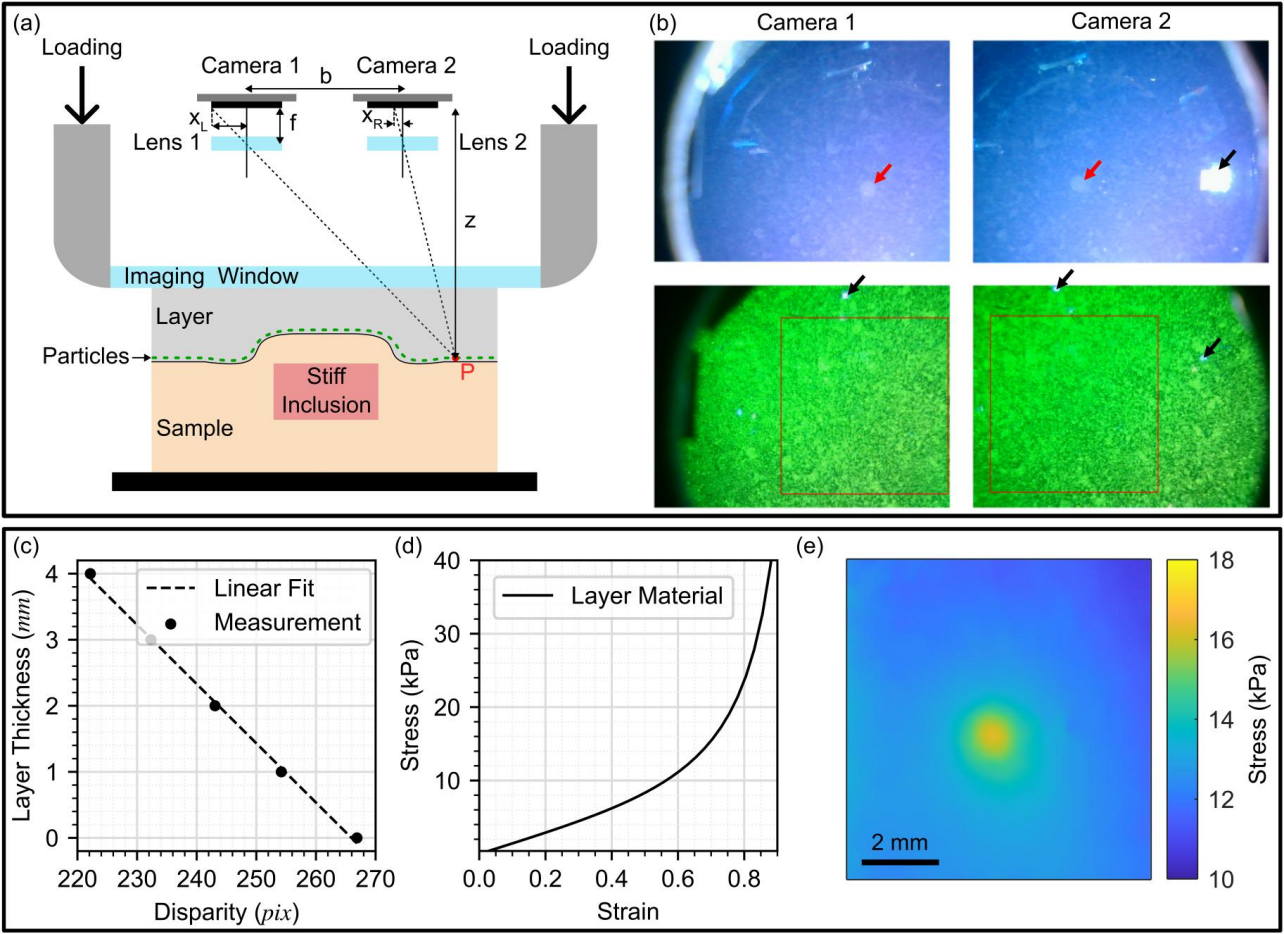
Working principle of SOP. Disparity between features in the UV illuminated phosphorescent particle layer images is used to measure the compressed thickness of the layer. The original thickness of the layer is used to estimate strain, then a mechanical model of the layer allows conversion to the final stress image. (a) Probe schematic showing the principles of stereoscopy used to relate disparity to distance. (b) White light and UV illuminated images from each camera, with red arrows indicating the location of the inclusion and black arrows indicating the location of visual artifacts from LED reflections. (c) Linear fit used to estimate layer thickness from disparity, a separate fit exists for each pixel location. (d) Neo-Hookean mechanical model of the layer material generated from uniaxial compression testing, which allows conversion from strain to stress. (e) Stress image generated by applying the mechanical model in (d) to strain derived from the total layer thickness change.

The probe-acquired camera images under white light and UV light illuminations, as shown in Fig. 4(b), were transferred to a laptop over Wi-Fi and a custom software method was used to calculate disparity, which is the lateral shift in pixels between identical features in images from the parallel cameras. Similar to the benchtop configuration of SOP,^33^ the images were undistorted and rectified using OpenCV's inbuilt stereoscopic calibration methods, which required data from ∼50 images of a checkerboard grid at different distances and orientations to the camera. For each pixel in our FOV, in the left image, the location of the corresponding pixel in the right image was found by calculating a normalized least squares difference cost function between a 35-pixel diameter circular window centered on the pixel of interest in the left image, and a range of potential matching circles in the right image near the expected location. The random pattern of phosphorescent particles in the camera images under UV light illumination created unique windows with a large number of features, reducing the noise that would be present and window size that would be required if using only the sparsely featured white light images for disparity calculation. Differing from the benchtop implementation, the lowest cost match and its neighbors were fit to a quadratic by which we calculated the sub-pixel disparity between the two images at this point, allowing a non-discretized estimation of disparity. This was repeated for each pixel within a rectangular region of the overlapped FOV. When running this algorithm on a NVIDIA GeForce GTX 1650 Ti graphics card, a frame was generated every 0.34 s. The frame rate could be increased with superior hardware and software optimization. For live display, while acquiring data, we processed only every second pixel in *x* and *y* with an appropriately smaller window size, enabling video rate processing.

The calculated disparity image is related to the distance from the cameras to the particles at the bottom of the layer via the principles of stereoscopy, as shown in Fig. 4(a) and as described in detail previously.^33^ The distance from the camera plane to the particle layer, z, is calculated as

$$z=\frac{b\;f}{x_{L}-x_{R}},$$ (1)

where b is the distance between the centers of the camera sensors, f is the focal length of the lenses, and $x_{L}-x_{R}$ is the disparity in units of distance. Here, z is the sum of the distances between multiple components and through materials with different refractive indexes, which are fixed apart from the layer thickness. Rather than measure the components of z directly, it is more convenient to empirically characterize the distance from the window to the bottom of the layer via a series of linear fits of disparity (in pixels) vs layer thickness, as seen in Fig. 4(c). These were generated at each pixel location by taking images of a 5 mm thick layer compressed by a flat plane parallel to the imaging window at distances of 1–4 mm. The 0 mm point was generated by compressing the side of the layer containing the particles directly against the imaging window. The benefit of performing these fits at each pixel position, rather than as an average over the image as per the benchtop implementation,^33^ is that we also calibrated for the tilt between the camera plane and the imaging window, and any residual errors from the undistortion of the camera images. Additionally, we measured the change in FOV as the imaging window decreased in distance to the sample surface from 4 to 0 mm, resulting in a maximum overlapping region of 10.7 × 8.4 mm^2^ (at 4 mm) and 8.9 × 6.9 mm^2^ (at 0 mm). The number of pixels per millimeter varied almost linearly over this range and, as such, a linear fit was used to generate a function from which to calculate the approximate FOV given the average distance from the window to the bottom of the layer over the image. This approach was used to generate the scale bars in all optical palpation images presented.

The distance image, d(x,y), was then converted to layer strain, $\epsilon_{E}\left(x,y\right)$, using the known uncompressed thickness of the layer, $L_{0}$, calculated as

$$\epsilon_{E}\left(x,y\right)=\frac{L_{0}-d(x,y)}{L_{0}}.$$ (2)

Stress was estimated from strain using the pre-characterized stress–strain curve shown in Fig. 4(d), which is a Neo-Hookean hyperelastic material model fit to data averaged from three uniaxial compression tests of the layer material. We refer to this approach as *the algebraic method* of calculating stress. In SOP images, stress is presented as true stress ( $\sigma_{T}$), calculated as

$$\sigma_{T}\left(x,y\right)=\sigma_{E}\left(x,y\right)\times \left(1+\epsilon_{E}\left(x,y\right)\right),$$ (3)

where $\sigma_{E}$ is the engineering stress and $\epsilon_{E}$ the engineering strain. This facilitates comparison with the results from the computational method described in Sec. V C, which are output from ABAQUS in the form of true stress.

### B. Computational optical palpation

**FIG. 5.**
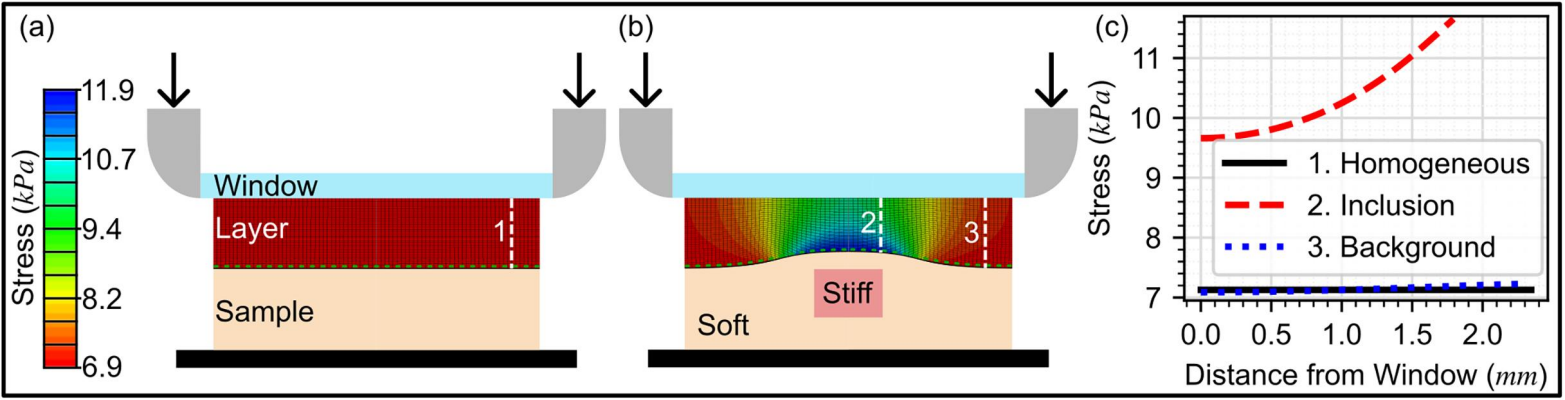
FEA derived stress distribution within the layer under frictionless compression of (a) a mechanically homogeneous flat sample, (b) a soft sample background with a stiff inclusion. (c) A plot of axial stress with distance at each of three locations marked with dashed white lines and numbered in (a) and (b), showing the changes in stress from the top of the layer to the bottom.

To convert the layer axial strain to stress, optical palpation assumes that friction at the layer boundaries is negligible and that stress in the layer is uniaxial and constant with depth. Figure 5(a) shows an FEA simulation of the stress distribution in the layer under these assumptions in the case of a homogeneous sample. These are common assumptions in many forms of elastography; however, they result in a less accurate mechanical model that reduces resolution and contrast in optical palpation images.^37^ Figure 5(b) shows an FEA simulation of the layer stress distribution when a stiff inclusion is embedded in the sample. In this case, the mechanical heterogeneity in the sample causes a change in the previously uniform deformation of the layer. This change leads to a breakdown in the assumptions of uniaxial and constant stress with depth in the layer, where we observe high stress concentrated in the area nearest to the inclusion and gradual reduction in stress away from this area. Figure 5(c) highlights this by showing the stress along the white, dashed lines indicated as 1, 2, and 3 in Figs. 5(a) and 5(b), which show how the stress varies in the axial direction. Under real experimental conditions, it is challenging to completely remove the effects of friction between every contact surface. The effects of friction, as well as boundary conditions related to the geometry of the layer and probe, cause further deviations from the assumptions.

Computational optical palpation is a method designed to address these challenges, originally implemented for use in OCT-based optical palpation by Wijesinghe *et al.*^35^ It uses a finite element model, implemented in ABAQUS 2020 (Dassault Systèmes, France), of the imaging window, layer, and sample surface. The model simulates the compression of the layer between the imaging window and the sample surface, using the information from the layer deformation measured by OCT to move the elements of the sample surface to a matching position in the simulation. This model avoids the assumption of uniaxial stress that is constant with depth in the layer, as each element in the layer experiences mechanical stress that depends on the deformation of that element in the simulated compression, and the mechanical model of the layer material, which was characterized using uniaxial compression tests. The stress at the layer–sample interface is extracted by retrieving the stress from the elements of the layer in contact with the simulated sample surface. Additionally, computational optical palpation allows for the simulation of friction during compression. Wijesinghe *et al.* characterized states of low, medium, and high friction depending on the amount of lubricant used between surfaces. As such, computational optical palpation provides a more accurate mechanical model, removing the assumptions of uniaxial stress, constant stress with depth, and frictionless contact, which allows for more accurate and higher resolution stress images.

To recreate this improvement in our work, we have adapted computational optical palpation^35^ to incorporate the experimentally measured layer deformation, along with the probe and layer geometry, into a numerical model, utilizing FEA to accurately estimate the stress distribution at the tissue surface. This method uses ABAQUS to simulate the deformation of the layer by placing it between the probe tip and a representation of the sample surface, which moves from an uncompressed state to a state matching the layer displacement measured experimentally using SOP. Key differences in the use of this method between the original benchtop OCT-based optical palpation and our handheld SOP probe implementation include the layer thickness increasing from ∼500 *μ*m to ∼5 mm, and the expected average strain in the layer increasing from ∼10% to ∼40%. To allow for larger strains compared to the previously published computational method, we represent the layer material with a Neo-Hookean mechanical model, add enhanced hourglass control to elements, and alter the initial element dimensions to have an aspect ratio closer to unity after compression. Additionally, to account for changes in stress brought about by changing boundary conditions of the layer, in our implementation we simulate the entire layer and model the geometry of the probe surrounding the imaging window. In the original implementation, only the imaged region is simulated, and the effect of boundary conditions is ignored. We also use the “low” friction coefficient of 0.2, characterized by Wijesinghe *et al.*, corresponding to the layer being covered with lubricant. The adapted ABAQUS FEA model and associated MATLAB and Python code used in this article are publicly available at https://github.com/RhysJonesResearch/A-wireless-and-handheld-optical-palpation-imaging-probe-for-use-in-breast-conserving-surgery.

### C. Layer and phantom fabrication

To manufacture the silicone layer, which is required to be transparent under white light illumination and emit patterns corresponding to the layer surface under UV illumination, 3D printed molds for cylindrical layers of diameter 19 mm and thickness 5.0 mm were used. The molds were filled with silicone (Wacker Elastosil, P7676, Parts A and B) at a 1:1 mixing ratio (Young's modulus ∼17 kPa at 10% strain). Once cured and removed, phosphorescent microparticles (Glowing Gecko, diameter < 30 *μ*m) were randomly distributed on one surface, and compressed against it. The layers were then placed, particles down, in a container with a larger diameter (>50 mm) with a thin (∼0.2 mm) layer of uncured silicone of the same mixture and weighted to compress the layers into the mixture. This compression displaced most of the mixture beneath the layer and resulted in the particles being embedded in the layer less than 50 *μ*m from the surface, which was determined using an OCT system (Thorlabs Telesto).

Phantoms for testing the performance of the probe were manufactured using a two-part 3D printed mold. The first part was cylindrical with a central pillar with dimensions corresponding to the size of the required inclusion, and thickness chosen to control how deep the inclusion was embedded in the phantom, and was filled with Ecoflex 00-20 part A, part B and silicone thinner (Smooth-On) at a ratio of 1:1:1 (tangent modulus ∼15 kPa at 10% strain). The void resulting from the pillar, once cured, was filled with RT601 part A and B (Wacker) at a ratio 5:1 (tangent modulus ∼900 kPa at 10% strain) to make the inclusion. The second cylindrical part spanned the full thickness of the phantom, with a slightly larger diameter to allow the cured silicone from the first part to fit in it. This mold section was also filled with Ecoflex 00-20 in the same ratio. The inclusion phantom in Fig. 2 was manufactured to have a 35 mm diameter and 3.5 mm thickness with an inclusion of diameter 0.8 mm and thickness 2 mm, 0.3 mm below the surface.

### D. Clinical testing

To test the device in a clinical setting, we imaged four human breast specimens freshly excised from three mastectomy surgeries at Fiona Stanley Hospital, Western Australia. The cases included one benign specimen, one spindle cell carcinoma specimen (from which we received two samples at different locations), and one invasive ductal carcinoma (IDC) specimen. Samples were excised by a pathologist in ∼30 × 35 × 5 mm blocks. Saline was used to provide lubrication between the tissue and the layer, while AK50 silicone oil (Wacker) was used between the layer and the imaging window of the probe. The tissue was compressed to the point where low stress regions were between 5 and 10 kPa, monitored using the live-view functionality of the probe software. After imaging, the edges of the samples were stained with ink to provide orientation in histology and the samples embedded in paraffin. Histology slides were sectioned in the same plane as the stress images and stained with hematoxylin and eosin. Co-registration of optical palpation images to histology images was performed by overlapping scaled images of the histology images to match macroscopic photographs of the samples. Individual regions of interest were then obtained by matching the probe's white light photographs with regions on the macroscopic photograph. This study was approved and ethics obtained as described in the Ethics Approval section below.

## SUPPLEMENTARY MATERIAL

See the supplementary material for additional details on the design of the custom circuit boards used in the probe and on the 3D printed case design.

## Data Availability

The data that support the findings of this study are available from the corresponding author upon reasonable request and custom models and code are openly available in GitHub at https://github.com/RhysJonesResearch/A-wireless-and-handheld-optical-palpation-imaging-probe-for-use-in-breast-conserving-surgery, Ref. 51.
